# *Sargassum pallidum* reduces inflammation to exert antidepressant effect by regulating intestinal microbiome and ERK1/2/P38 signaling pathway

**DOI:** 10.3389/fphar.2024.1424834

**Published:** 2024-07-18

**Authors:** Dan Su, Qianmin Li, Xin Lai, Yonggui Song, Huizhen Li, Zhifu Ai, Qi Zhang, Wenxiang Shao, Ming Yang, Genhua Zhu

**Affiliations:** ^1^ Key Laboratory of Evaluation of Traditional Chinese Medicine Efficcacy (Prevention and Treatment of Brain Disease with Mental Disorders), Key Laboratory of Depression Animal Model Based on TCM Syndrome, Jiangxi Administration of Traditional Chinese Medicine, Jiangxi University of Chinese Medicine, Nanchang, China; ^2^ Jiangxi Guxiang Jinyun Comprehensive Health Industry Co. Ltd., Nanchang, China

**Keywords:** sargassum pallidum, network pharmacology, inflammation, antidepressant, gut microbiota

## Abstract

Immune inflammation is one of the main factors in the pathogenesis of depression. It is an effective and active way to find more safe and effective anti-inflammatory depressant drugs from plant drugs. The purpose of this study is to explore the potential of marine plant *Sargassum pallidum (Turn).C.Ag.* (Haihaozi, HHZ) in the prevention and treatment of depression and to explain the related mechanism. Phytochemical analysis showed that alkaloids, terpenes, and organic acids are the main constituents. *In vitro* and *in vivo* activity studies showed the anti-neuroinflammatory and antidepressant effect of *Sargassum pallidum*, furthermore, confirmed that 7-Hydroxycoumarin, Scoparone, and Kaurenoic Acid are important plant metabolites in Sargasum pallidum for anti-neuroinflammation. Mechanism exploration showed that inhibition of ERK1/2/p38 inflammatory signaling pathway contributing to the antidepressant effect of *Sargassum pallidum* in reducing intestinal inflammatory levels. This study confirmed the value of *Sargassum pallidum* and its rich plant metabolites in anti-inflammatory depression, providing a new choice for the follow-up research and development of antidepressant drugs.

## 1 Introduction

Depression is a serious and complex mental disorder, which has become a major killer of human beings. The pathogenesis of depression is very complex, including monoamine depletion, neuroinflammation, genetic and epigenetic abnormalities, neuroendocrine hypotheses, *etc.* Among them, the monoamine depletion hypothesis has always dominated the pathophysiology of depression (Uchida et al., 2018).

At present, the first-line drugs for the treatment of depression:SSRI drugs, As classic monoamine drug, which can cause dry mouth, constipation, orthostatic dizziness, in clinical practice, their long-term use can easily lead to blurred vision and anticholinergic adverse reactions (Uher et al., 2009). With the continuous accumulation of clinical immunological evidence, the research and development of depression prevention and treatment drugs have extended from the excitation of monoamine neurotransmitter neurons to the regulation of central inflammatory immunity (Beurel et al., 2020). Studies have shown that nonsteroidal anti-inflammatory drugs (NSAIDs) can significantly improve depressive symptom (Zuzarte et al., 2018), yet Long term use of non steroidal anti-inflammatory drugs may cause a series of adverse reactions such as nausea, vomiting, gastric ulcers, hypertension, and acute kidney injury (Theken et al., 2020). Due to depression is a chronic disease requiring long-term medication, the safety of long-term medication is the focus of clinical treatment selection and application. Therefore, there is an urgent need to find more safety and less side effect depression prevention and treatment drugs.

Immune inflammation has been proved to be one of the main factors in the pathogenesis of depression, and the development of inflammation is closely related to depression (Rajkowska and Stockmeier, 2013). The imbalance of microorganisms in the body will lead to the release of inflammatory factors increases, and influenced depressive symptoms occur (Beurel et al., 2020). Therefore, regulating the microbiota for the treatment of depression has been received increasing attention (Chudzik et al., 2021). Modern research analysis showed that plant metabolites can improve intestinal microbiota imbalance and immune disorders (Hu et al., 2021; Li et al., 2021). Thus, exploration more phytomedicines in regulating intestinal flora and preventing inflammatory is a promising approach for depression treatment. Among a large number of plant medicines, marine plants are an important component that cannot be ignored. Marine plant drugs contain a large amount of active metabolites, which are widely distributed and have high yields, and are also receiving increasing attention.

The traditional Chinese medicine “haizao (*Sargassum pallidum*)” was first recorded in the “Shennong Materia Medica Classic” and is classified as a medium grade. It has the effects of reducing phlegm, softening hardness, dispersing nodules, promoting diuresis, and reducing swelling. Sargasum is a wild plant resource with high development, utilization, and medicinal value. It has the advantages of rich active ingredients, wide distribution, abundant resources (Premarathna et al., 2020). Modern pharmacology indicates that Sargassum is rich in plant metabolites such as alginate, fucoidan sulfate, and taurine, and has various physiological activities such as antioxidant, anti-tumor, and anti gastric ulcer properties (Chen et al., 2022). The medicinal plants of the genus Sargassum have a wide range of anti-inflammatory activities, some of which also have anti neuroinflammatory effects (Han et al., 2021; Liyanage et al., 2022). Considering the high correlation between neuroinflammation and depression, it is necessary to explored whether Sargasum pallidum has the potential to treat depression, as well as the specific mechanisms of anti-inflammatory depression.

In this article, we used UHPLC-QTOF-MS/MS technology to complete the analysis of plant metabolites contained in the *Sargassum pallidum*, obtaining its chemical profile. The animal model of inflammatory depression induced by LPS and the neuroinflammation model of BV2 cells were selected to carry out pharmacodynamic studies *in vivo* and *in vitro*, based on them, the antidepressant effects of seaweed and its plant metabolites were explored. At the same time, it is found that the gut microbiota and its inhibitory ERK1/2/p38 inflammatory signaling pathway play a crucial role in improving depression. This study confirmed the potential of *Sargasum pallidum* and its rich plant metabolites in anti-inflammatory depression. It also emphasized the important value of phytomedicines in regulating intestinal microbiome and reducing inflammation to play an antidepressant role.

## 2 Materials and methods

### 2.1 Consumables and chemicals

*Sargassum pallidum(Turn).C.Ag.* (Haihaozi, HHZ) (batch number: 19061309) was purchased from Anguo Yifang Pharmaceutical Co., Ltd. (Anguo, China) and was identified as HHZ dried whole herb by Wu Shuyao who is a Chinese pharmacist of Qihuang National Medical College, Jiangxi University of Chinese Medicine. Chemicals and their suppliers are summarized in Supplementary Table S1 of Supplementary Methods.

### 2.2 Preparation of seaweed

Accurately weigh 500 g HHZ of dried algal bodies, crush them and put them in a 10000 mL round bottom flask. Add 10 times the amount of 80% ethanol (5000 mL) as the solvent, condensing reflux extraction for 8 h, 5 times the amount of solvent was added to the residue for reflux extraction 1 h. The twice obtained extracts mixed was rotary evaporated to remove ethanol, and the remaining concentrated solution was freeze-dried for 24 h. The HHZ extract is dried by a freeze-drying machine and ground into freeze-dried powder, which is stored in a dryer away from light for better storage.

### 2.3 UHPLC-QTOF-MS/MS analysis

Accurately weigh 1 g of medicinal material powder was weighed and mixed with ten times the amount of 80% methanol (10 mL), followed by ultrasonic extraction for 60 min. The liquid phase and mass spectrometry analysis parameters are shown in Supplementary Table S2.

### 2.4 Cell lines and culture

The BV2 microglial cell line was obtained from the Cell Bank of the Chinese Academy of Sciences (Shanghai, China). BV2 cells were maintained in complete DMEM medium containing 10% fetal bovine serum. All cells were incubated at 37°C in a humidified atmosphere of 5% CO_2_.

#### 2.4.1 Cell viability assay

BV2 cells were seeded into 96-well plates at an initial density of 6×10^3^ cells/well. The cultured cells were divided into Control group, Model group, Positive drug (PAR) group, and different dosage groups. The Control group was not treated, while the remaining groups were added with LPS at a final concentration of 1 μg/mL. HHZ extracts at final concentrations of 20 μg/mL, 40 μg/mL, 60 μg/mL, 80 μg/mL, and 100 μg/mL were added. The final concentrations of 7-hydroxycoumarin (7-OH), Scoparone (BH), and Kaurenoic Acid (YBK) were all 10 μM, and the PAR group was added with paroxetine at a final concentration of 5 μM. Add 10 μL of CCK-8 solution to each well. After incubation for 1.5 h, read the OD value at 450 nm using a microplate reader.

#### 2.4.2 Preparation of medicated serum

54 healthy SPF C57BL/6J were randomly divided into control group and treatment groups. Administer paroxetine group and HHZ plant metabolites group 10 mg/kg, HHZ low, medium, and high doses of HHZ 100 mg/kg, 200 mg/kg, and 300 mg/kg, respectively by gavage, the control group was given an equal volume of physiological saline for five consecutive days. After intraperitoneal injection of pentobarbital anesthesia, blood was collected and centrifuged at 4°C at 3,000 r/min for 15 min. The upper serum was separated, inactivated in a water bath, and packaged with a 0.22 μm filter membrane. It was stored at −80°C for later use.

#### 2.4.3 ELISA

Used Elisa reagent to detect the levels of inflammatory cytokines interleukin-6 (IL-6), NO, IL-4, interleukin-1 β (IL-1 β), and tumor necrosis factor alpha (TNF-α) in BV2 cells.

#### 2.4.4 RT-qPCR

Collect total RNA from BV2 cells and colon tissue using Trizol reagent. Use a transcription kit to reverse RNA into cDNA. The iNOS qPCR primer sequence is upstream:GGCTGCCCCTGGAAGT, downstream:TGCAAGT-GAAATCCGATTGG, and the ZO-1 primer sequence is upstream:CCATCTTGGACGATTGTG, downstream:TAATGCCCGAGCTCCGATG.

### 2.5 Animal experiments

#### 2.5.1 Animals and drug administration

Male C57BL/6J mice (6°weeks old) were purchased from Hunan Slack Jingda Co., Ltd. Before the experiment, all the mice were exposed to an SPF room at 20°C–22°C, at humidity of 45%–65%, with 12 h light/dark cycle, free access to water and food. The animal experiment protocol was approved by the Animal Ethics Committee of Jiangxi University of Chinese Medicine (Nanchang, China) on 9 November 2021 (Approval Number: JZLLSC20210061). After 1 week of adaptive feeding, the mice were randomly divided into control group, model group, paroxetine positive group 10 mg/kg, HHZ high-dose group 300 mg/kg, HHZ medium-dose group 200 mg/kg, HHZ low-dose group 100 mg/kg, 10 mg/kg of 7-OH group, 10 mg/kg of YBK group, and 10 mg/kg of BH group, eight mice in each group.

#### 2.5.2 LPS injection combined with orphanage method to establish inflammatory depression model and behavioral evaluation

Except for the control group, the other groups were injected with LPS continuously for 5°days, 1 mg/kg once a day. In addition, the model group and treatment group mice were raised in a quiet environment with a single cage, and the padding was changed once a week. The control group mice were raised in the same environment with four mice per cage. The plant metabolites group was administered by intraperitoneal injection, the positive drug group and the HHZ extract were administered by gavage. Depression-like behavior was evaluated by sucrose preference test (SPT), open field test (OFT), elevated plus maze test (EPM) and tail suspension test (TST). All ethology procedures refer to literature and previous work (Song et al., 2021; Pei et al., 2024).

#### 2.5.3 16S r RNA gene sequencing and data analysis

Fresh stool samples were collected in sterile cryovials on the same day and stored in a −80°C freezer until use. The DNA of the fecal samples was extracted and then subjected to Polymerase Chain Reaction (PCR) amplification and product purification. Shanghai Meiji Biomedical Technology Co., Ltd. was authorized to use the MiseqPE300 platform for microbiome analysis.

#### 2.5.4 HE staining

Intestinal tissues were rinsed three times with Phosphate Buffered Saline (PBS), fixed in 4% paraformaldehyde, embedded in paraffin, and made into 5 μM sections. Then sections were deparaffinized with xylene and varying concentrations of ethanol. After HE staining, the pathological changes of intestinal tissue were observed under light microscope.

#### 2.5.5 Immunofluorescence

BV2 cells were placed in confocal dishes, colon paraffin sections (5 μM), and deparaffinized with different concentrations of xylene and ethanol. Antigen retrieval was performed in warmed citrate buffer (10 mM, pH 6.0). Block with goat serum for 1 h at room temperature, use the following primary antibodies: rabbit anti-IL-6 (1:500), rabbit anti-TNF-α (1:500), IL-1β (1:250) rabbit anti-phospho-ERK1/2 (1:25), rabbit anti-phospho-p38-MAPK (1:50). Then store overnight in a 4°C refrigerator. Secondary antibody was added after washing: goat anti-rabbit IgG (H + l) (1 h at room temperature), after addition of 4′,6-Diamidino-2-Phenylindole (DAPI) for nuclear visualization, anti-fluorescent quencher was also dropped on the slide. Images were analyzed by Leica Software and Fiji (ImageJ).

### 2.6 Statistical analysis

All data analysis was performed using the GraphPad Prism 8.0 data analysis software. All data are presented as mean ± SD. One-way analysis of variance was used for comparisons involving more than three groups, *t*-test were used for comparisons involving two groups. *p*-value <0.05 was considered statistically significant.

## 3 Results

### 3.1 Phytochemical composition

**FIGURE 1:**
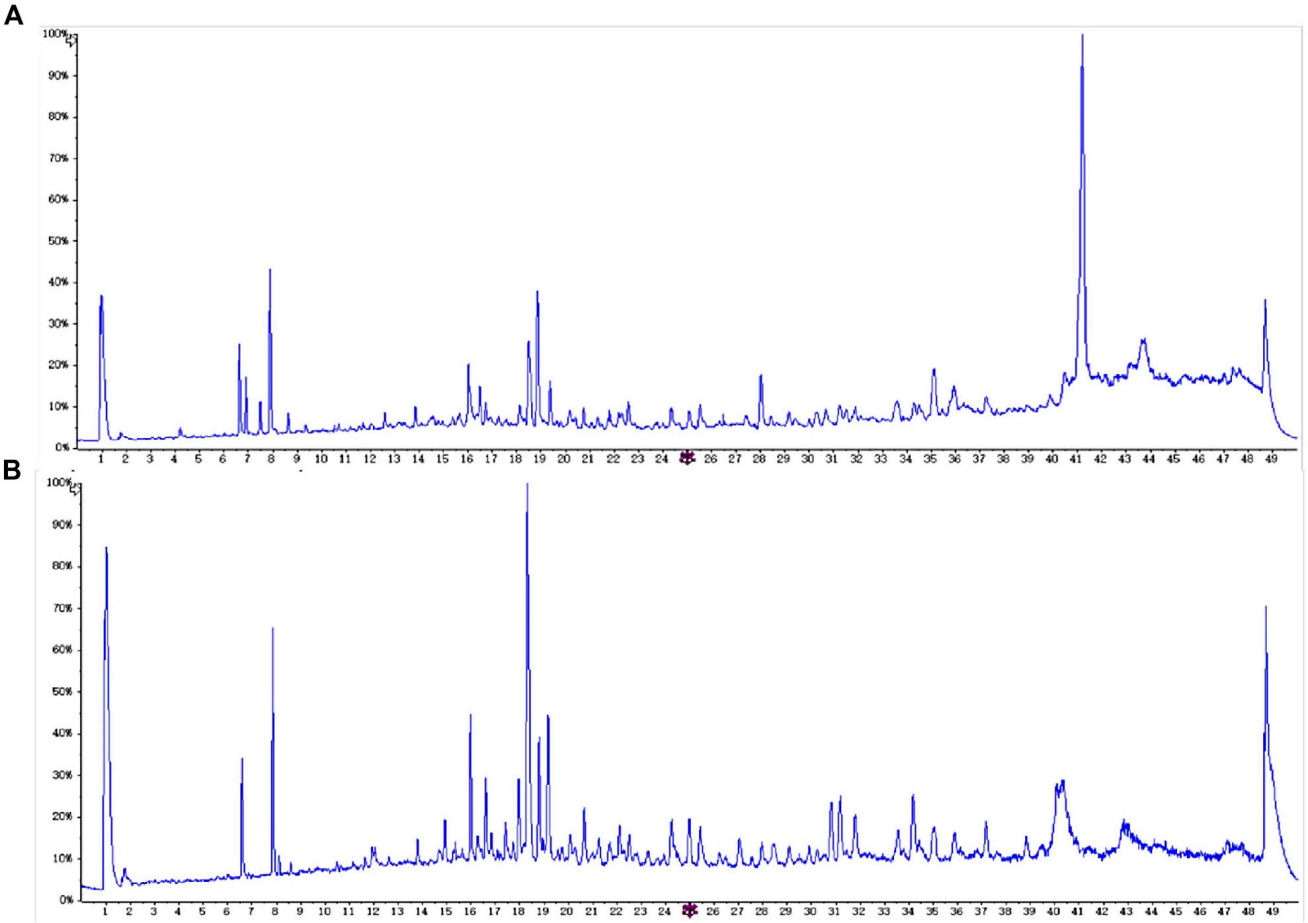
HHZ chemical composition analysis, **(A)** positive ion mode, **(B)** negative ion mode.

The samples were detected by UHPLC-QTOF-MS/MS, and the obtained data were analyzed using Analyst TF 1.6 and peakview1.2 data processing system (Sciex Corporation). Through mass spectrum fragment ion analysis, mass spectrum database matching and comparison of relevant literature reports, a total of 45 chemical components were identified (Figure 1; Supplementary Table S3). Compared with the composition analysis results of *Sargassum fusiforme (Harv.)Setch.* (YXC) in our research group before, it was found that the amount of alkaloids, glycosides, and lignans in HHZ was higher than that in YXC (Supplementary Figure S4). Comparing the relative content of various metabolites in two types of seaweed, we found that the content of phenylpropanoid and saponins in HHZ was higher. Coumarins rich in HHZ are the main characteristic compounds. (Supplementary Figure S5). These data indicate that HHZ has more advantages in composition and development value than YXC.

### 3.2 Protective effect of HHZ on BV2 cell inflammation induced by LPS

**FIGURE 2:**
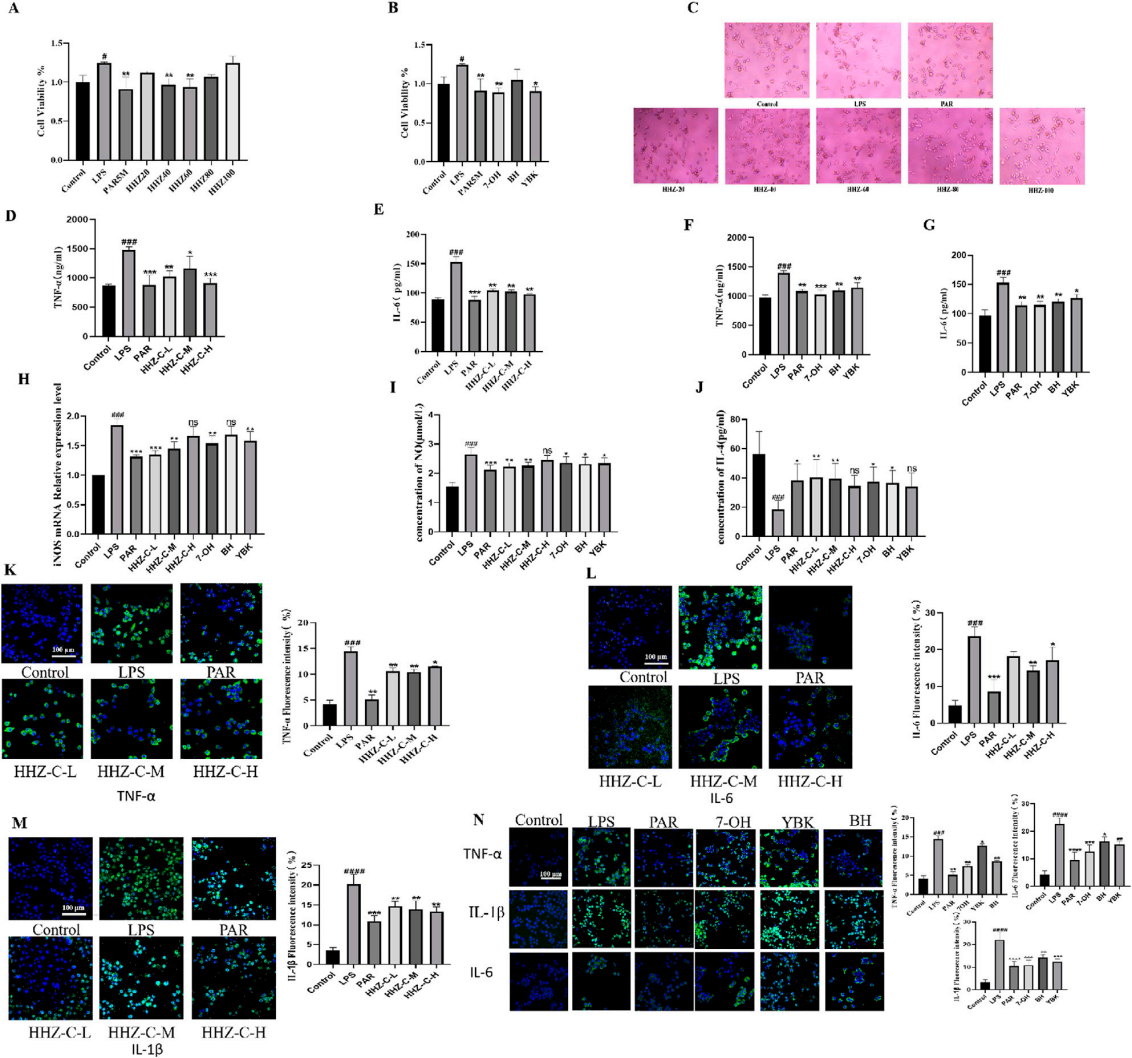
Protective effect of HHZ on LPS-induced inflammation in BV2 cells. **(A, B)** CCK-8 cell viability detection of HHZ extract and its active ingredient. **(C)** The effect of HHZ extract and LPS on the morphology of BV2 microglial cells (100X). **(D–G)** ELISA detection of TNF-α and IL-6 levels in BV2 cells. **(H–J)** After treatment with medicated serum, the expression levels of iNOS, NO, and IL-4 in BV2 cells. **(K–N)** Immunofluorescence analysis of TNF-α, IL-6, IL-1β. Values represent the mean ± SD. #*p* < 0.05, ##*p* < 0.01, ###*p* < 0.001, ####*p* < 0.0001 means significant difference compared with the control group; *p < 0.05, **p < 0.01, ***p < 0.001, ****p < 0.0001 means significant difference compared with LPS group.

The results of CCK-8 showed that in the positive drug group, paroxetine could effectively inhibit the activity changes induced by LPS. The HHZ extract inhibited the activation of microglial cells in a dose-dependent manner, and the other 7-OH, BH, and YBK groups decreased the cell activation index after administration, while the inhibitory effect of the BH group was weak. The cell viability of each of the above groups was insignificantly reduced, which also suggested that the administration groups did not produce cytotoxic effects (Figures 2A, B). The difference in cell morphology was observed under the microscope. Most of the normal BV2 cells in the control group were relatively round, with a small cell body diameter, and synaptic growth of cells with longer morphology was not seen. The number of activated cells in the model group treated with LPS increased notably, and the cell body became hypertrophic and the cell synapses grew. The number of activated cells in the administration groups of 20 μg/mL and 100 μg/mL extracts of seaweed was more, and the number of activated cells increased remarkably, while the number of activated cells in other groups was relatively small (Figure 2C). In summary, 40 μg/mL, 60 μg/mL, and 80 μg/mL seaweed extract can be selected as the subsequent experimental concentration.

In order to further verify the anti-neuroinflammatory effect of HHZ extract and its plant metabolites, we detected the contents of TNF-α and IL-6 of microglial inflammatory factors in each group (Pei et al., 2023). It was found that the contents of TNF-α and IL-6 in the supernatant of BV2 cells in the Model group increased considerably, and different doses of administration could reduce the concentrations of these two inflammatory factors to varying degrees (Figures 2D–G). Additionally, to determine whether HHZ and its plant metabolites inhibit the inflammatory response of BV2 cells and whether it is related to phenotype polarization, we detected the expression levels of M1 phenotype markers iNOS mRNA and NO in BV2 cells and the content of M2 phenotype marker IL-4 in drug-containing serum. The results showed that compared with the control group, the expression of iNOS mRNA and NO was significantly increased after LPS stimulation, while the content of IL-4 was significantly reduced. After treatment with drug-containing serum, these indicators all showed varying degrees of improvement (Figures 2H–J). Then we measured the inflammatory factors TNF-α, IL-1β, and IL-6 by cell immunofluorescence experiments, and it showed that administration of low, medium and high doses of HHZ extracts could all make the levels of LPS-induced cellular inflammatory factor TNF -α, IL-1β, IL-6 decreased. In the experiment, the level of IL-6 in the low-dose HHZ group showed a certain degree of decrease. Among the three groups, the middle-dose one (60 μg/mL) had best effect (Figures 2K–M). The three monomers of 7-OH, BH and YBK also showed good anti-neuroinflammatory effects, among which 7-OH had the best anti-inflammatory activity (Figure 2N). These data indicate that HHZ and its active ingredients exhibit excellent anti-neuroinflammatory activity and neuroprotective effect in cells *in vitro*. For the purpose of further validating the anti-inflammatory activity and neuroprotective effect of HHZ and its plant metabolites *in vivo*, we used the mouse model of LPS-induced inflammation leading to depression to explore the mechanism of action of HHZ against inflammatory depression.

### 3.3 Improvement effect of HHZ and its plant metabolites on LPS-induced depression-like mice

**FIGURE 3:**
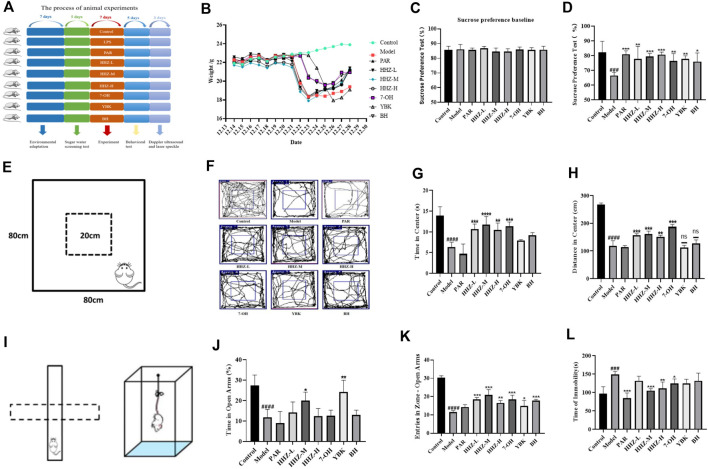
Improvement effect of HHZ and its active ingredients on LPS-induced depression-like mice. **(A)** Experimental design. **(B)** The trend of body weight of mice in each group. **(C–D)** Effects of HHZ and its active components on anhedonia in depressed mice in SPT. **(E–H)** Effects of HHZ and its active components on spontaneous locomotion in depressed mice in OFT. **(I–K)** Effects of HHZ and its active components on exploratory behavior in depressed mice in EPM. **(L)** Effects of HHZ and its active components on immobility time in depressed mice in TST. Values represent the mean ± SD. #*p* < 0.05, ##*p* < 0.01, ###*p* < 0.001 ####*p* < 0.0001 means significant difference compared with Control group; *p < 0.05, **p < 0.01, ***p < 0.001, ****p < 0.0001 means significant difference compared with Model group.

Firstly, the body weight changes of the mice in each group were evaluated, and it was found that the body weight of the mice in the Model group was obviously lower than that of the mice in the control group. However, after administration, none of the HHZ dose groups and their active components showed significant changes in mouse body weight (Figure 3B). Mice were evaluated using four behavioral tests: SPT, OFT, EPM, and TST. Compared with the control group, the mice in the Model group had an apparently lower preference for sucrose, and this trend was reversed after treatment with HHZ and its active ingredient (Figures 3C, D). In the OFT, the Model mice showed overt decrease in locomotor activity, the state recovered after treatment with HHZ and its active ingredients. We noticed that moderate doses of HHZ produced a pretty stable effect (Figures 3E–H). In the EPM, compared with the Control group, the percentage of time spent in the open arm and the number of open arm entries were reduced massively in the model group mice, all doses of HHZ and plant metabolites administration groups were able to significantly increase the number of times they entered the open arm, and HHZ-M and YBK groups were also able to significantly increase the stay time of the open arm (Figures 3I–K). And in the TST, the immobilization time of model mice was longer than that of control mice, and the immobility time after treatment with HHZ and its plant metabolites was clearly shorter than that of model group. Among them, the medium dose of HHZ showed the best effect (Figure 3L).

### 3.4 Intestinal inflammation improvement of HHZ in depression mice

**FIGURE 4:**
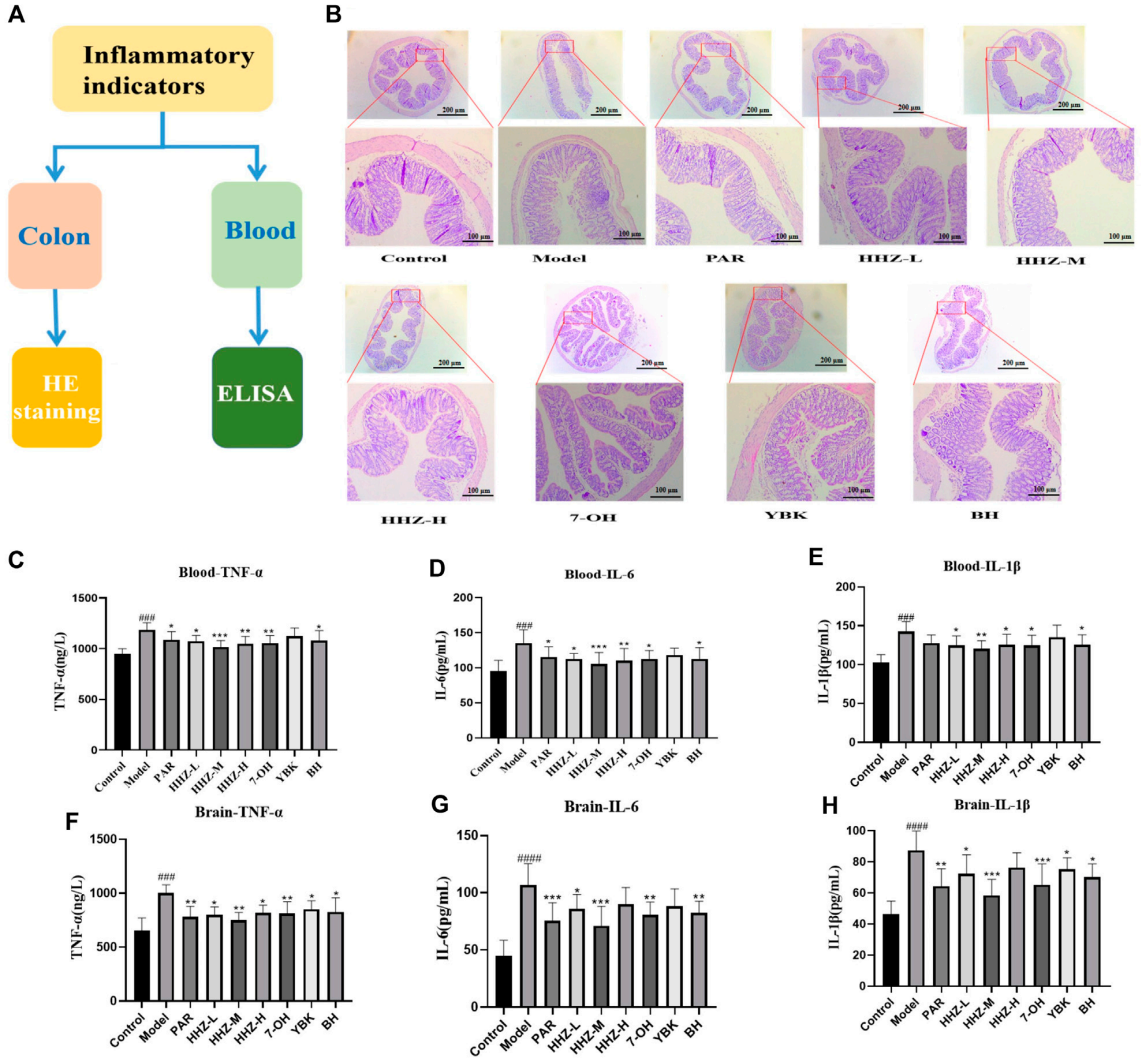
The improvement effect of HHZ on the intestinal environment of inflammatory depression mice. **(A)** Experimental design. **(B)** Pathological examination of mouse colons in each group by HE staining. **(C–E)** Elisa detection of serum TNF-α, IL-6, IL-1β levels of mice in each group. **(F–H)** Elisa detection of Brain TNF-α, IL-6, IL-1β levels of mice in each group. Values represent the mean ± SD. #*p* < 0.05, ##*p* < 0.01, ###*p* < 0.001 ####*p* < 0.0001 means significant difference compared with Control group; *p < 0.05, **p < 0.01, ***p < 0.001, ****p < 0.0001 means significant difference compared with Model group.

The intestinal environment can affect human psychology and behavior by regulating brain development and behavioral patterns through the brain-gut axis. Therefore, we first used HE staining to perform pathological examination on the colon tissues of mice in each group. The pictures showed that the goblet cells in the control group were in complete shape, the crypts were arranged neatly, and there was no conspicuous inflammatory infiltration. After LPS modeling, goblet cell morphocytes and crypts were severely lost in the model group, and obvious inflammatory infiltration appeared at the same time. By administering HHZ and its active ingredients in each group, except for the control group and the model group, the intestinal inflammation of the mice in each group showed varying degrees of recovery, among which HHZ-L, HHZ-M, 7-OH, and BH very well improved the inflammatory state of mice in the model group, and the cell morphology in the other groups was mostly intact (Figure 4B). Then we used ELISA to measure the content of serum and brain inflammatory factors TNF-α, IL-1β, and IL-6 in inflammatory depression mice, and found that HHZ and its three active ingredients can greatly reduce the content of these three inflammatory factors (Figures 4C–H), which show that HHZ and its plant metabolites may exert antidepressant effects by improving intestinal inflammation.

**FIGURE 5:**
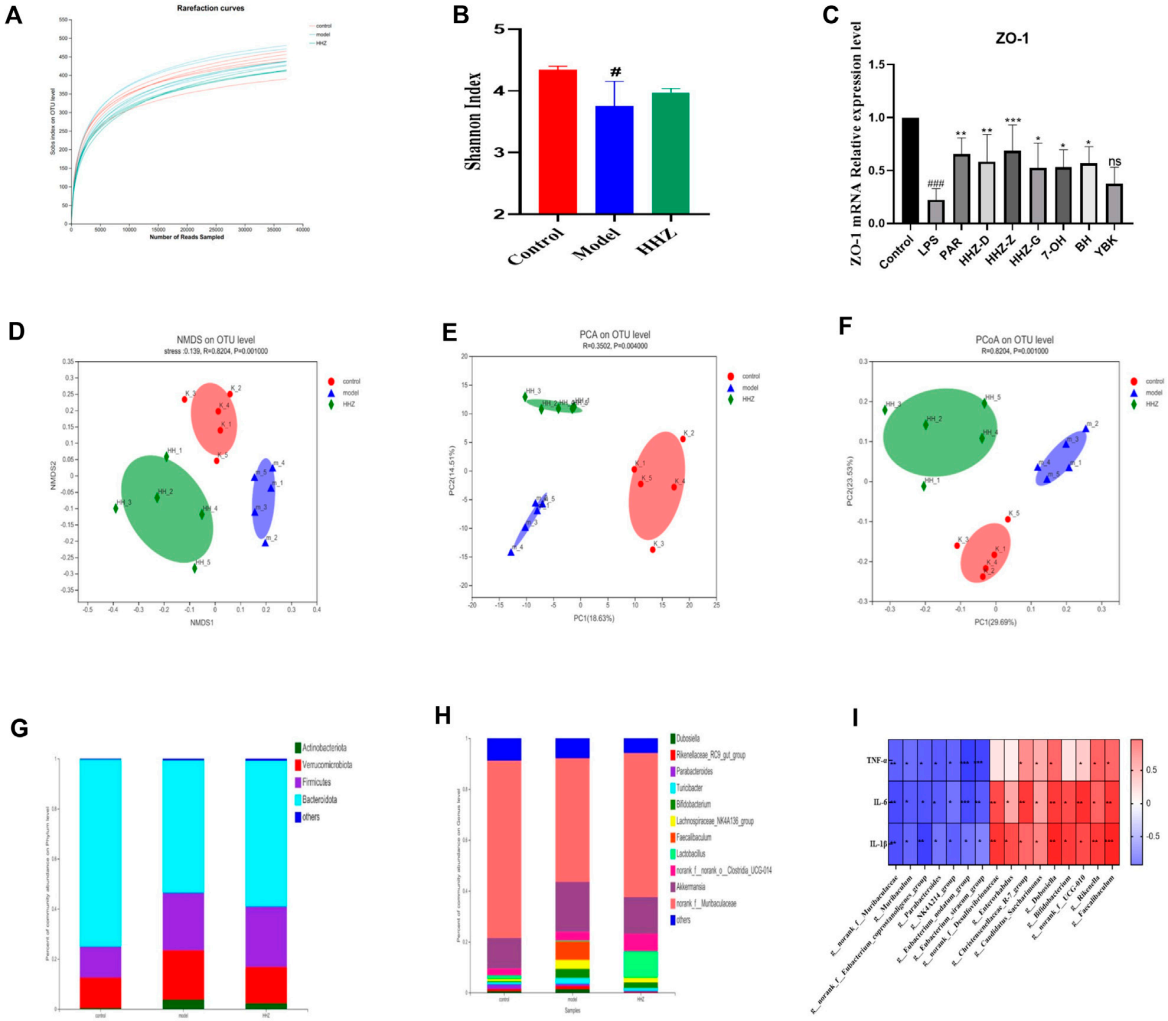
The improvement effect of HHZ on the gut microbiota of inflammatory depression mice. **(A–B)** Effects of HHZ on microbial alpha diversity sobs and shannon indices. **(C)** Relative expression of ZO-1 gene in intestinal tissue. **(D)** NMDS method to analyze species differences in microbial communities. **(E)** PCA analysis of gut microbiota β-diversity. **(F)** Differences in gut microbiota between different groups in the PCoA study. **(G)** Differences in microbial communities in feces at the phylum level. **(H)** Distribution of microbial communities in feces at the genus level. **(I)** Correlation between different levels of Spearman’s gut bacteria and identification of depression-related inflammatory indices. Values are presented as mean ± SD. #*p* < 0.05 means a significant difference compared with the control group, and the significance of the relevant results is represented by *p < 0.05, **p < 0.01, ***p < 0.001.

We collected fecal samples from mice in the control group, model group, and HHZ gavage group for 16s RNA sequencing analysis to further explore the impact of HHZ on the intestinal environment, and the obtained Sobs index and Shannon index had no difference between the groups (Figures 5A, B). PCA analysis showed that the β-diversity of the model group was mighty different from that of the control group, while HHZ partially reversed this change (Figures 5D–F), suggesting that HHZ can regulate the diversity of gut microbiota. At the level of phylum classification, compared with the Control group, the richness of Bacteroidota went down, and the richness of Actinobacteriota, Verrucomicrobiota, and Firmicutes rose in the Model group, while the HHZ group reduced the increase of these three bacterial phyla and alleviated the decrease in the richness of Bacteroidota (Figure 5G). At the level of genus classification, compared with the control group, the proportion of norank_ f_ _Muribaculaceae richness in the Model group was distinctly reduced, and the richness of Akkermansia, Bifdobacterium, Lachnospiraceae and NK4A136 group increased. It is worth noting that the HHZ group can recover the richness of norank_ f_ _Muribaculaceae, reduce the proportion of Akkermansia, Bifdobacterium, Lachnospiraceae and NK4A136 group, and greatly increase the richness of Lactobaillus (Figure 5H). The correlation between intestinal flora and serum inflammatory factors was verified by spearman correlation analysis, and the results showed that there was a close relationship between intestinal bacteria and inflammatory factors. Among these bacterial genera, beneficial bacterial genera such as g-norank, f-Muribaculaceae, and g-Muribaculum had a strong negative correlation with the levels of IL-1β, IL-6, and TNF-α, while g-Faecalibaculum, g-Dubosiella, and IL-1β, IL-6, and TNF-α levels showed a strong positive correlation (Figure 5I). In addition, we also used RT qPCR to detect the mRNA level of intestinal barrier protein ZO-1, and the results showed that compared with the control group, the mRNA expression level of ZO-1 in the Model group was significantly reduced. After administration, the expression of ZO-1 mRNA showed varying degrees of regression (Figure 5C). These data suggested that HHZ exerts antidepressant effects by improving the gut microbiota structure in inflammatory depressed mice.

### 3.5 Inflammatory inhibition of HHZ on ERK1/2/P38 signaling pathway

**FIGURE 6:**
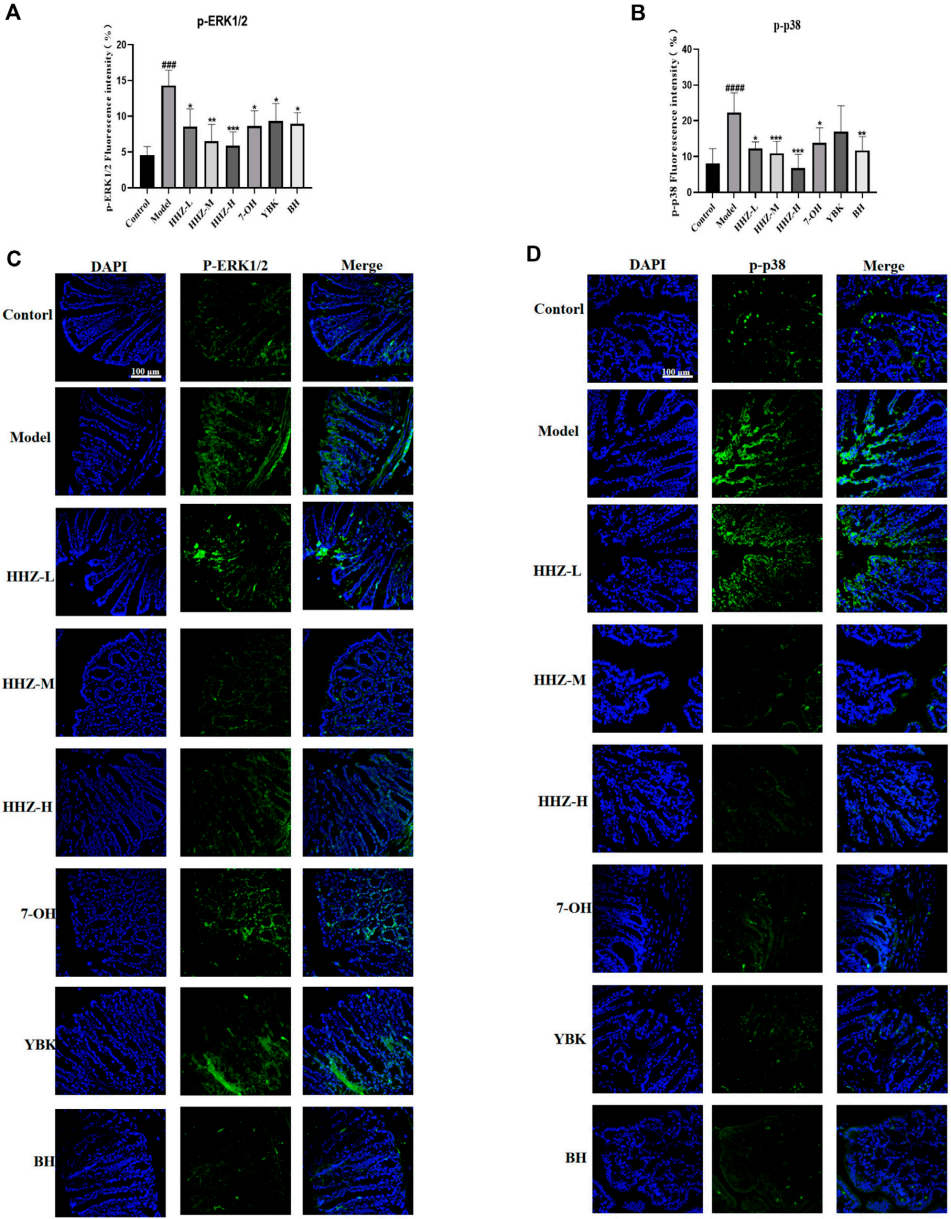
Gut microbiota-based antidepressant mechanism of HHZ involves expression inhibition of ERK1/2/P38 signaling pathway. **(A, C)** Immunofluorescent staining of p-ERK1/2 in the colon. **(B, D)** Immunofluorescent staining of p-p38 in the colon. Values represent the mean ± SD. #*p* < 0.05, ##*p* < 0.01, ###*p* < 0.001 ####*p* < 0.0001 means significant difference compared with Control group; *p < 0.05, **p < 0.01, ***p < 0.001, ****p < 0.0001 means significant difference compared with Model group.

Since HHZ can regulate lipopolysaccharide (LPS)-producing intestinal bacteria and inflammatory factors, it is suggesting that the anti-inflammatory depressive effect of HHZ may be related to the ERK1/2/P38 signaling pathway, which is a key and usually stimulated by pro-inflammatory factors (Chahwan et al., 2019). The expressions of p-ERK1/2 and p-p38 were detected in the colon tissue of mice in each group by immunofluorescence method. The results showed that the expression level of p-ERK1/2/p-p38 in model group mice was markedly higher than that in control group mice, while HHZ and its plant metabolites could reverse this trend (Figures 6A–D). It can be seen that HHZ can inhibit the activation of ERK1/2/P38 signaling pathway, and the potential anti-inflammatory depression mechanism of HHZ and its plant metabolites is related to the transmission of ERK1/2/P38 signaling pathway mediated by intestinal flora.

## 4 Discussion

Many different dosing strategies have been adopted in the past decade to increase the efficacy of conventional antidepressants (Chenu et al., 2006), but clinical responses remain unsatisfactory (Lam et al., 2002), and as many as One-third of depressed patients still are resistant to treatment with these conventional antidepressants (Wohleb et al., 2016). With the further deepening of the research on marine drugs, more and more algae have been developed from simple consumption to medical services. A large number of studies have proved that marine algae have anti-inflammatory, anti-tumor, antibacterial and other effects. In this study, *Sargassum pallidum* and its plant metabolites showed good anti-inflammatory depressive activity, which indicated that the comprehensive utilization of marine algae had a bright prospect.

We established a UHPLC-QTOF-MS/MS analysis method, and identified main plant metabolites of seaweed, mainly alkaloid, terpenoids and coumarins, and also rich in vitamins and amino acids. LPS-induced microglial activation can lead to an inflammatory response and applied in cellular models (Shen et al., 2022; Zhai et al., 2023). Therefore, in this study, LPS (lipopolysaccharide) was used to stimulate BV2 microglia to establish an *in vitro* neuronal inflammation model, and to verify the anti-neuroinflammatory effect of *Sargassum pallidum* and its three plant metabolites. Using the CCK-8 kit to measure the viability of microglial cells, it was found that LPS increased the activity of microglial cells, indicating that the model was successful. The generation of neuroinflammation is inseparable from high levels of inflammatory factors (Han et al., 2024). The levels of IL-1β, TNF-α, and IL-6 were measured by enzyme-linked immunosorbent assay and immunofluorescence assay, among them, the medium dose HHZ group (60 μg/mL) can ensure the highest cell activity while reduced the concentration of the three inflammatory factors mentioned above and avoided the induction of neuroinflammation and neurotoxicity by abnormally activated microglia. This experiment proved that *Sargassum pallidum* and its plant metabolites can inhibit the increase of the secretion of pro-inflammatory factors. It showed that *Sargassum pallidum* and its plant metabolites have anti-inflammatory activity, may have antidepressant activity, and may also have the potential to treat other central diseases caused by inflammation.

*In vitro*, *Sargassum pallidum* extract can effectively reduce the production of inflammatory factors and protect nerve cells. *In vivo*, *Sargassum pallidum* improve the depression-like behavior of chronic inflammation-induced depression mice. Specifically, after administration, the immobility time in the FST and the TST reduced, while sugar water preference increased in the SPT. The results of *in vitro* and *in vivo* pharmacological effects show that 7-OH, BH, and YBK are important metabolites of the anti-inflammatory and depressive effects of *Sargassum pallidum*.

In the LPS induced depression model, the intestine showed a significant inflammatory response. HE staining of the intestinal tissue verified its pathological changes. The inflammatory factors in the blood have a direct impact on the level of inflammation in the brain, meanwhile also regulated by the level of intestinal inflammation (Wang et al., 2022; Liu et al., 2023). Therefore, the expression levels of inflammatory factors in serum and brain tissue of each group were further detected. The results showed that the expression levels of inflammatory factors in the serum and brain tissue of the model group were significantly increased, at the same time, *Sargassum pallidum* and its active ingredients could improve inflammation to varying degrees The gut microbiota plays an important role in the occurrence and development of depression, and the imbalance of the microbiota will directly affect the rate of neuroinflammation. In the analysis and comparison of the taxonomic levels of the microbiota in each group of mice, we found that the model group had a significant decrease in the abundance of beneficial bacterial genera norank_ f_ Mauribaulaceae and Lactobacaillus, and a significant increase in the abundance of harmful bacterial genera Akkermansia, Bifdobacterium, Lachnospiraceae and NK4A136. *Sargassum pallidum* could significantly increase the number of norank_ f_ Mauribaulaceae and reduce the abundance of Bifdobacterium, La4A136. The quantity of chnospiraceae and NK4A136 is worth noting that HHZ can greatly increase the richness of *Lactobacillus*, *Lactobacillus* is the largest genus of lactic acid bacteria, which not only enhances the intestinal mucosa and the systemic immune system to exert anti-inflammatory effects, but also improves the structure of the intestinal microbiota community by regulating intestinal microbiota metabolism, and exerts antidepressant effects through the brain gut microbiota axis. This may be an important pathway for *Sargassum pallidum* to produce good antidepressant effects.

In order to explore the mechanism of *Sargassum pallidum* reducing intestinal inflammation while regulating the flora, we explored potential ERK1/2/P38 pathway using immunofluorescence. It was found that *Sargassum pallidum* can regulate the ERK1/2/p38 signaling pathway, inhibit the phosphorylation of ERK1/2/p38 protein, and reduce the release of intestinal inflammatory factors. Meanwhile, 7-OH, BH and YBK can also control the inflammatory factors of BV2 microglial cells *in vitro* by regulating the ERK1/2/p38 signaling pathway. Studies have shown that ERK1/2 and p38 signals participate in the regulation of the secretion and release of inflammatory factors in the inflammatory stress of cells, and regulate the differentiation and apoptosis of cells in stress (Sun et al., 2015). This indicates that HHZ affects the balance of the intestinal microenvironment, improves intestinal immunity, and transmits signals through the central nervous system along the brain gut axis, thereby reducing neuroinflammation which can alleviate the development of depression.

## 5 Conclusion

This study used LC-MS to analyze the material composition of *Sargassum pallidum*, and found that coumarins are one of the main characteristic plant metabolites. At the same time, combining the *in vivo* and *in vitro* activity studies of LPS inflammatory depression model and BV2 cells, not only confirmed the antidepressant effect of *Sargassum pallidum*, but also exhibited that 7-OH, BH, and YBK having anti neuroinflammation effect, which can significantly reduce and alleviate the inflammatory activation of microglia, and significantly improve the depressive behavior of inflammatory depression mice. Finally, based on 16S rDNA microbiota technology, combined with immunofluorescence staining, it was confirmed that the ERK 1/2/p38 inflammatory signaling pathway is the key pathway for alleviating inflammatory depression in *Sargassum pallidum*. This study may provide valuable insights into the discovery of new therapeutic uses for traditional medicinal plants, and also suggest that *Sargassum* may be a valuable plant for inflammatory or psychiatric diseases.

## Data Availability

The original contributions presented in the study are included in the article/Supplementary Material, further inquiries can be directed to the corresponding author.

## Abbreviation

BH, Scoparone; CUR, Curtain Gas; DAPI, 4′,6-Diamidino-2-Phenylindole; DV, Declustering Voltage; EDTA, Ethylene Diamine Tetraacetic Acid; EPM, Elevated Plus Maze Test; GS1, Atomizing Gas; GS2, Auxiliary Heating Gas; HHZ, Sargassum pallidum; IL-6, Interleukin-6; IL-1β, Interleukin-1β; OFT, Open Field Test; 7-OH, 7-Hydroxycoumarin; PBS, Phosphate Buffered Saline; PCR, Polymerase Chain Reaction; SPT, Sucrose Preference Test; TNF-α, Tumor Necrosis Factor-α; TST, Tail Suspension Test; YBK, Kaurenoic Acid.
