# Correction: *Sargassum pallidum* reduces inflammation to exert antidepressant effect by regulating intestinal microbiome and ERK1/2/P38 signaling pathway

**DOI:** 10.3389/fphar.2025.1653830

**Published:** 2025-10-14

**Authors:** Dan Su, Qianmin Li, Xin Lai, Yonggui Song, Huizhen Li, Zhifu Ai, Qi Zhang, Wenxiang Shao, Ming Yang, Genhua Zhu

**Affiliations:** ^1^ Key Laboratory of Evaluation of Traditional Chinese Medicine Efficcacy (Prevention and Treatment of Brain Disease with Mental Disorders), Key Laboratory of Depression Animal Model Based on TCM Syndrome, Jiangxi Administration of Traditional Chinese Medicine, Jiangxi University of Chinese Medicine, Nanchang, China; ^2^ Jiangxi Guxiang Jinyun Comprehensive Health Industry Co. Ltd., Nanchang, China

**Keywords:** sargassum pallidum, network pharmacology, inflammation, antidepressant, gut microbiota

There was a mistake in Figure 2 as published. The cell morphology images of the “Control group”, “Model group”, and “PAR group” in Figure 2C were accidentally misused due to confusion in the naming of the experimental archive files, using some images from previous studies of the research group under the same experimental conditions. The immunofluorescence images of the “IL-1β Control group” and “IL-6 Control group” in Figure 2N were mistakenly pasted with the same control group image due to file classification errors during image export. The corrected Figure 2 appears below.

**FIGURE 2:**
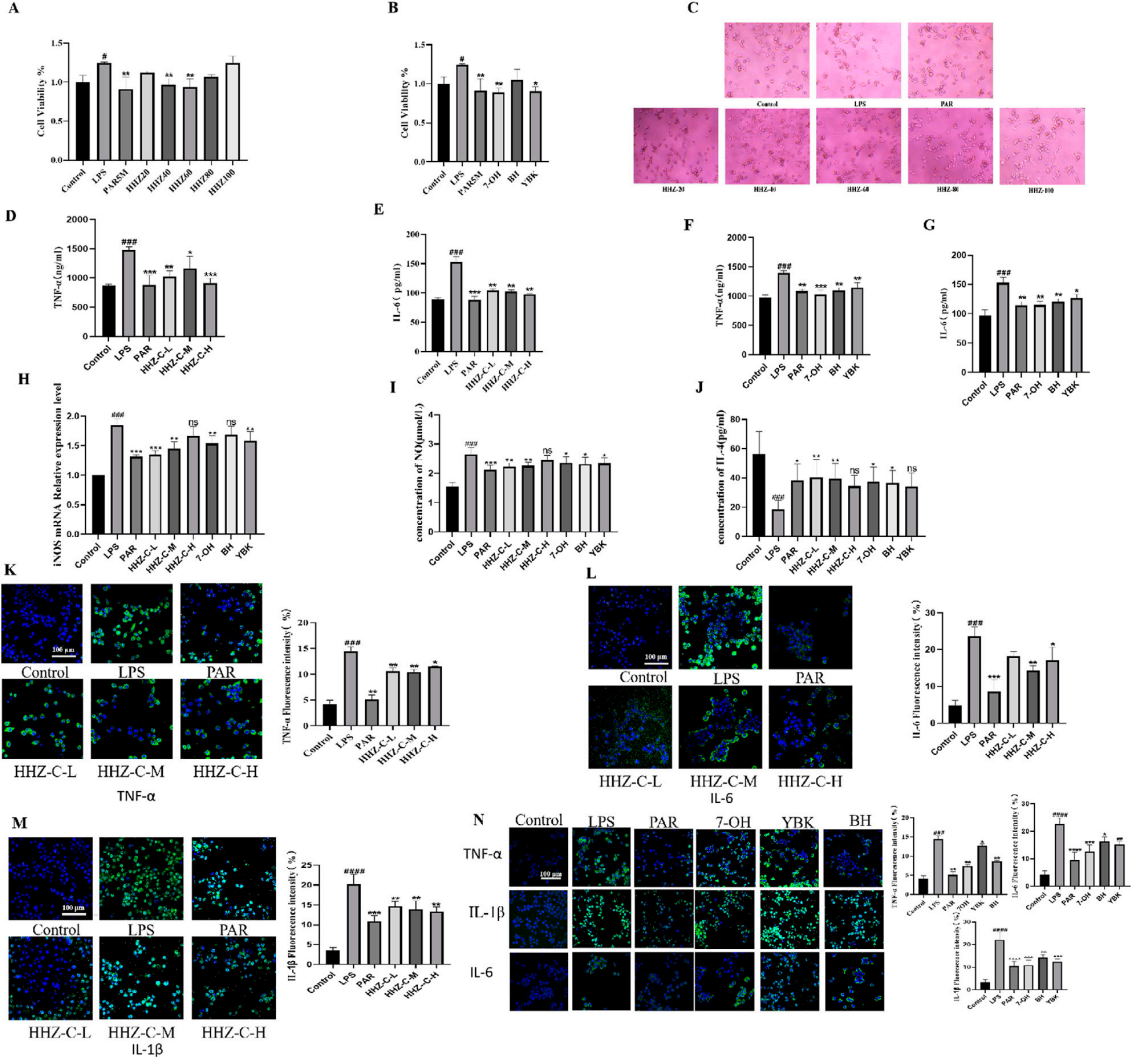
Protective effect of HHZ on LPS-induced inflammation in BV2 cells. **(A,B)** CCK-8 cell viability detection of HHZ extract and its active ingredient. **(C)** The effect of HHZ extract and LPS on the morphology of BV2 microglial cells (100X). **(D–G)** ELISA detection of TNF-α and IL-6 levels in BV2 cells. **(H–J)** After treatment with medicated serum, the expression levels of iNOS, NO, and IL-4 in BV2 cells. (**K–N**) Immunofluorescence analysis of TNF-α, IL-6, IL-1β. Values represent the mean ± SD. #p < 0.05, ##p < 0.01, ###p < 0.001, ####p < 0.0001 means significant difference compared with the control group; *p < 0.05, **p < 0.01, ***p < 0.001, ****p < 0.0001 means significant difference compared with LPS group.

The original article has been updated.

