# Supplementary material for: Sargassum pallidum reduces inflammation to exert antidepressant effect by regulating intestinal microbiome and ERK1/2/P38 signaling pathway
Source: Front Pharmacol. 2024 Jul 18;15:1424834. doi: 10.3389/fphar.2024.1424834 (PMC11291328; doi:10.3389/fphar.2024.1424834)
Supplement: Supplementary file 1 [file DataSheet1.docx]

**Supporting Information**

***Sargassum pallidum* reduces inflammatory response to exert antidepressant effect by regulating intestinal microbial structure and ERK1/2/P38 signaling pathway**

Dan Su ^1,a^, Qianmin Li^1,a^, Xin Lai^1^, Yonggui Song^1^, Huizhen Li^1^, Zhifu Ai^1^, Qi Zhang^1^, Wenxiang Shao^1^, Ming Yang^1,2^, Genhua Zhu^1,*^

*^1^Key Laboratory of Evaluation of Traditional Chinese Medicine Efficcacy(Prevention and Treatment of Brain Disease with Mental Disorders), Key Laboratory of Depression Animal Model Based on TCM syndrome, Jiangxi Administration of Traditional Chinese Medicine, Jiangxi Province, Jiangxi University of Chinese Medicine, 1688 Meiling Road, Nanchang 330006, China.*

*^2^ Jiangxi Guxiang Jinyun Comprehensive Health Industry Co., Ltd., Nanchang, China*

**^[[1]](#footnote-1)^**

**Table S1 Reagents**

| Test Drugs/Reagents | Number | Manufacturer |
| --- | --- | --- |
| Paroxetine | Y0000578 | Sigma-Aldrich (Shanghai) Trading Co., Ltd. |
| Methanol | F112084 | ACS Corporation |
| Acetonitrile | AS1122-801 | TEDIA Corporation |
| Ethyl Acetate | 3952 | Tianjin Damao Chemical Reagent Factory |
| Kaurenoic acid | AB0300 | Alpha Biological Company (Chengdu, China) |
| 7-Hydroxycoumarin | AB0072 | Alpha Biological Company (Chengdu, China) |
| Nobiletin | AB0864 | Alpha Biological Company (Chengdu, China) |
| Scoparone | AB1701 | Alpha Biological Company (Chengdu, China) |
| Hematoxylin | D1005-1-4 | Nanjing Jiancheng Technology Co., Ltd. (Nanjing, China) |
| Eosin dye | D1006-1-4 | Nanjing Jiancheng Technology Co., Ltd. (Nanjing, China) |
| Isoflurane | 792632-1G | ALDRICHCompany |
| PBS | CBS101.05 | Cellmax Corporation |
| High glucose DMEM medium | CGM102.05 | Cellmax Corporation |
| penicillin-streptomycin | C100C5 | Cellmax Corporation |
| Pancreatin | T1300 | Solarbio Corporation |
| CCK-8 Kit |  | GLPBIO Corporation |
| LPS | L2880 | Sigma Corporation |
| TBST buffer solution | T1082 | Solarbio Corporation |
| TritonX-100 | T8220 | Solarbio Corporation |
| BSA | A8020 | Solarbio Corporation |
| DAPI | TX01025 | Shanghai Yingxin Laboratory Equipment Co., Ltd. |
| TITC-goat anti-rabbit IgG | BA1105 | Boster Biological Co., Ltd. |

**Table S2 chromatography and mass spectrometry conditions**

| UPLC | |
| --- | --- |
| Column | Acquity UPLC BEH C18 chromatographic column (100 mm×2.1 mm, 1.7 μm, Waters company) |
| Column temperature（℃） | 40 |
| Mobile phase | A= 0.1% formic acid aqueous solution B= 0.1% formic acid acetonitrile |
| Gradient conditions | 0~15 min(5 %~55 %B), 15~45 min( 55 %~95 %B), 47~47.10min(95 %~5 %B) |
| Flow rate（mL/min） | 0.3 |
| Injection volume （uL） | 3 |
| Mass spectrometer | |
| Lon Source Temp （°C） | 500 |
| Declustering Voltage（V） | 100 |
| Voltage(V) | 4500 |
| Scan Range | 50~1550 |
| Curtain Gas(psi) | 40 |
| Atomizing gas and Auxiliary gas(psi) | 50 |
| Collision Energy(ev) | 40±10 |

**Table S3 UPLC-QTOF-MS/MS Identification Results of Compounds in *Sargassum pallidum***

| NO. | identity | Molecular formula | Found At RT (min) | Adduct | E.M (Da) | Error(ppm) | MS/MS | Types | **HHZ Original medicinal materials** | **YXC Original medicinal materials** | **HHZ**  **Freeze**  **dried**  **powder** |
| --- | --- | --- | --- | --- | --- | --- | --- | --- | --- | --- | --- |
| 1 | linoleic acid | C18H32O2 | 31.8 | -H | 279.233 0 | 2.5 | 261.2246 | OA | *√* | *√* | *√* |
| 2 | betaine | C5H11NO2 | 12.68 | +H | 118.086 3 | 2.8 | 59.07 0，58.063 | ALK | *√* | *√* | *√* |
| 3 | adenosine | C10H13N5O4 | 1.87 | +H | 268.104 | -0.8 | 136.0624，119.0350，57.0476 | ALK | *√* | *√* | *√* |
| 4 | adenine | C5H5N5 | 1.67 | +H | 136.0618 | 0.6 | 136.0618，119.0357，92.0261，65.0157 | ALK | *√* | *√* | *√* |
| 5 | protopine | C20H19NO5 | 8.29 | +H | 354.133 6 | 0.8 | 336.1247，275.0714，247.078，206.0826，188.0710，165.0560，149.0576 | ALK | *√* | *√* | *√* |
| 6 | l-carnitine | C7H15NO3 | 11.53 | +H | 162.112 5 | 0.5 | 102.0918，85.0297，60.0840，  58.0682 | ALK | *√* | *√* | *√* |
|  |  |  |  |  |  |  |  | ALK |  |  | *√* |
| 7 | uridine | C9H12N2O6 | 1.76 | -H | 243.0623 | 3.7 | 243.0213 | ALK | *√* | *√* |  |
| 8 | thymine | C5H6N2O2 | 2.02 | +H | 127.0502 | 2.1 | 110.0274，84.0466，82.0306，54.0389 | ALK | *√* | *√* | *√* |
| 9 | trichosanatine | C27H28N2O4 | 16.02 | +H | 445.2121 | 0.9 | 252.1035，224.1076，105.0345，91.0558 | ALK | *√* | *√* | *√* |
| 10 | sophoridine | C15H24N2O | 3.59 | +H | 249.1961 | 2.6 | 249.1962，231.1880，148.0776，136.1130，98.0982，96.0826，84.9616，79.0564，70.0678 | ALK | *√* |  | *√* |
| 11 | tetrahydropalmatine hydrochloride | C21H25NO4 | 8.85 | +H | 356.1856 | 1.1 | 356.1858，340.1550，192.1021，176.0712，165.0915，150.0673，148.0774 | ALK | *√* |  | *√* |
| 12 | 6-hydroxypurine | C5H4N4O | 1.74 | +H | 137.0458 | 1.5 | 137.0456，119.0360，110.0355，94.0406，82.0414，67.0309，65.0165，55.0333 | ALK | *√* |  | *√* |
| 13 | roburic acid | C30H48O2 | 38.6 | +H | 441.3726 | 0.6 | 441.3740，357.2881，245.1864，205.1936，189.1620，163.1483，149.0950，135.1173，107.0868 | TRI | *√* | *√* | *√* |
| 14 | 18β-glycyrrhetinic acid | C30H46O4 | 36.03 | -H | 469.3323 | -1 | 425.3408，407.3357，392.3089，381.3278 | TRI | *√* |  | *√* |
| 15 | 3-acetyl-11-keto-beta-boswellic acid | C32H48O5 | 16.33 | +H | 513.3574 | 0.3 | 513.3496，453.3395，435.3266，269.2296，201.1668，185.1357，149.0984，95.0870 | TRI | *√* |  | *√* |
| 16 | ambroxane | C16H28O | 30.31 | +H | 237.2213 | 0.2 | 149.1309，135.1186，121.1109，109.1008，95.0856，93.0707，81.0715，67.0562，55.0581 | DIT | *√* | *√* | *√* |
| 17 | sclareolide | C16H26O2 | 19.3 | +H | 251.200 6 | -0.3 | 233.1979，135.1164，121.1026，  107.086 6，95.0899，79.0548，67.0571 | DIT | *√* | *√* | *√* |
| 18 | kaurenoic acid | C20H30O2 | 22.2 | +H | 303.233 4 | 0.7 | 285.2220，257.1897，175.1483，  161.1318，147.1167，133.1012 | DIT | *√* | *√* | *√* |
| 19 | alpha-cyperone | C15H22O | 12.89 | +H | 219.1743 | -0.6 | 219.1738，145.1021，119.0873，105.0712，91.0556，77.0407，67.0599 | SE | *√* | *√* | *√* |
| 20 | atractylenolide iii | C15H20O3 | 11.75 | +H | 249.1485 | 0.3 | 170.1076，161.0950，155.0861，128.0627，105.0728，91.0545，79.0564，55.0211 | SE | *√* | *√* | *√* |
| 21 | curdione | C15H24O2 | 16.17 | +H | 237.1849 | -0.2 | 201.1675，159.1163，121.0996，105.0718，93.0721，77.0412，67.0561 | SE | *√* | *√* | *√* |
| 22 | parthenolide | C15H20O3 | 11.75 | +H | 249.1485 | 0.3 | 161.0950，142.0790，128.0627，105.0728，91.0545，81.0711，77.0421，55.0211 | SE | *√* | *√* | *√* |
| 23 | galangin | C15H10O5 | 16.64 | -H | 269.0456 | -3 | 269.0400，240.0400，225.0566，210.0345，181.0727 | FLA | *√* | *√* | *√* |
| 24 | apigenin | C15H10O5 | 16.63 | -H | 269.0456 | -1.3 | 269.0468，227.0341，195.0487，171.0428，167.0574，143.0458 | FLA | *√* | *√* |  |
| 25 | nobiletin | C21H22O8 | 13.97 | +H | 403.1388 | 0.7 | 403.1404，373.0936，327.0865，211.0258，183.0283 | FLA | *√* | *√* | *√* |
| 26 | cimifugin | C16H18O6 | 7.11 | +H | 307.1176 | 0.7 | 259.0603，235.0614，221.0451，205.0486，177.0543 | FLA | *√* |  |  |
| 27 | neoeriocitrin | C27H32O15 | 35.02 | -H | 595.1669 | 2 | 459.1838，339.1539 | FLA | *√* |  |  |
| 28 | riboflavin | C17H20N4O6 | 5.24 | -H | 375.131 | 0.8 | 255.0882，241.0716，212.0890 | VIT | *√* | *√* | *√* |
| 29 | retinoic acid | C20H28O2 | 19.8 | +H | 301.2162 | 1 | 283.2111，241.1948，199.1500，185.1328，173.1344，159.1168，145.1013，131.0858，117.0724，105.0713，91.0558，67.0568 | VIT | *√* | *√* | *√* |
| 30 | vitamin d2 | C28H44O | 46.73 | +H | 397.3465 | 0.7 | 379.3454，225.1626，201.1746，161.1329，147.1173，119.0875，109.0665，81.0730 | VIT | *√* | *√* | *√* |
| 31 | nicotinic acid | C6H5NO2 | 1.69 | +H | 124.0393 | 0.8 | 80.0515，78.0351，53.0421，50.0186 | VIT | *√* |  | *√* |
| 32 | ethyl 4'-hydroxy-3'-methoxycinnamate | C12H14O4 | 22.31 | +H | 223.0965 | -0.2 | 207.0321，149.0263，121.0295，91.0559，77.0387 | PHE | *√* | *√* | *√* |
| 33 | ethyl-p-coumaric acid (p) | C9H8O3 | 1.53 | +H | 165.0546 | 0.4 | 109.0653，79.0560，77.0412,51.0279 | PHE | *√* |  |  |
| 34 | 7-hydroxycoumarin | C9H6O3 | 10.86 | +H | 163.039 | 0.2 | 149.0223，121.0283，93.0341，65.0416 | COU | *√* | *√* | *√* |
| 35 | scoparone | C11H10O4 | 8.48 | +H | 207.0652 | -1 | 191.0339，163.0400，151.0757，107.0503，91.0556，77.0411，65.0413 | COU | *√* |  | *√* |
| 36 | salvianolic acid | C26H22O10 | 15.36 | +H | 495.1286 | -3.9 | 495.1282，195.0095，131.0877 | LIG | *√* |  | *√* |
| 37 | valine | C5H11NO2 | 1.07 | +H | 118.0863 | 2.8 | 118.0875，59.0770，58.0693 | AA | *√* | *√* | *√* |
| 38 | methyl linoleate | C19H34O2 | 33.99 | +H | 295.2632 | 0.8 | 263.2424，161.1301，133.1033，109.1015，81.0711，55.0601 | FA | *√* | *√* |  |
| 39 | sarsasapogenin | C27H44O3 | 39.92 | +H | 417.3363 | 0.7 | 417.3332，273.2204，161.1352，147.1177 | SAP | *√* | *√* | *√* |
| 40 | diosgenin | C27H42O3 | 35.9 | +H | 415.3207 | 0.9 | 415.2557，271.2077，253.1934，175.1498，147.1171，133.1011，119.0867 | SAP | *√* |  | *√* |
| 41 | muscone | C16H30O | 35.11 | +H | 239.2369 | -0.1 | 109.1014，95.0864，81.0728，67.0575，57.0737，55.0594 | Other | *√* | *√* | *√* |
| 42 | cis-13-docosenoamide | C22H43NO | 41.21 | +H | 338.3417 | 1.1 | 116.1089，97.0659，69.0730，57.0743 | Other | *√* | *√* | *√* |
| 43 | oleamide | C18H35NO | 30.69 | +H | 282.2791 | 0.8 | 111.0814，83.0868，69.0728，55.0581 | Other | *√* | *√* | *√* |
| 44 | (-)-umbelliferamide | C32H30N2O4 | 18.85 | +H | 507.2283 | 1 | 252.1021，224.1072，105.0324，91.0554，77.0406 | Other | *√* | *√* | *√* |
| 45 | -hydroxymethylfurfural | C6H6O3 | 3.63 | +H | 127.0389 | 1.6 | 127.0410，68.9982，59.9335 | Other | *√* |  | *√* |

OA: Organic acid, ALK: Alkaloids, TRI: Triperpenoids, DIT: Diterpenoids, SE: Sesquiterpenes, FLA: Flavonoids, QUI: Quinones, GLY: Glycoside, VIT: Vitamin, PHE: Phenylpropanoids, COU: Coumarin, LIG: Lignans, AA: Amino acid, FA: Fatty acid, SAP: saponin,

**Figure S1 Comparison of the amount of ingredients in various types of HHZ and YXC**


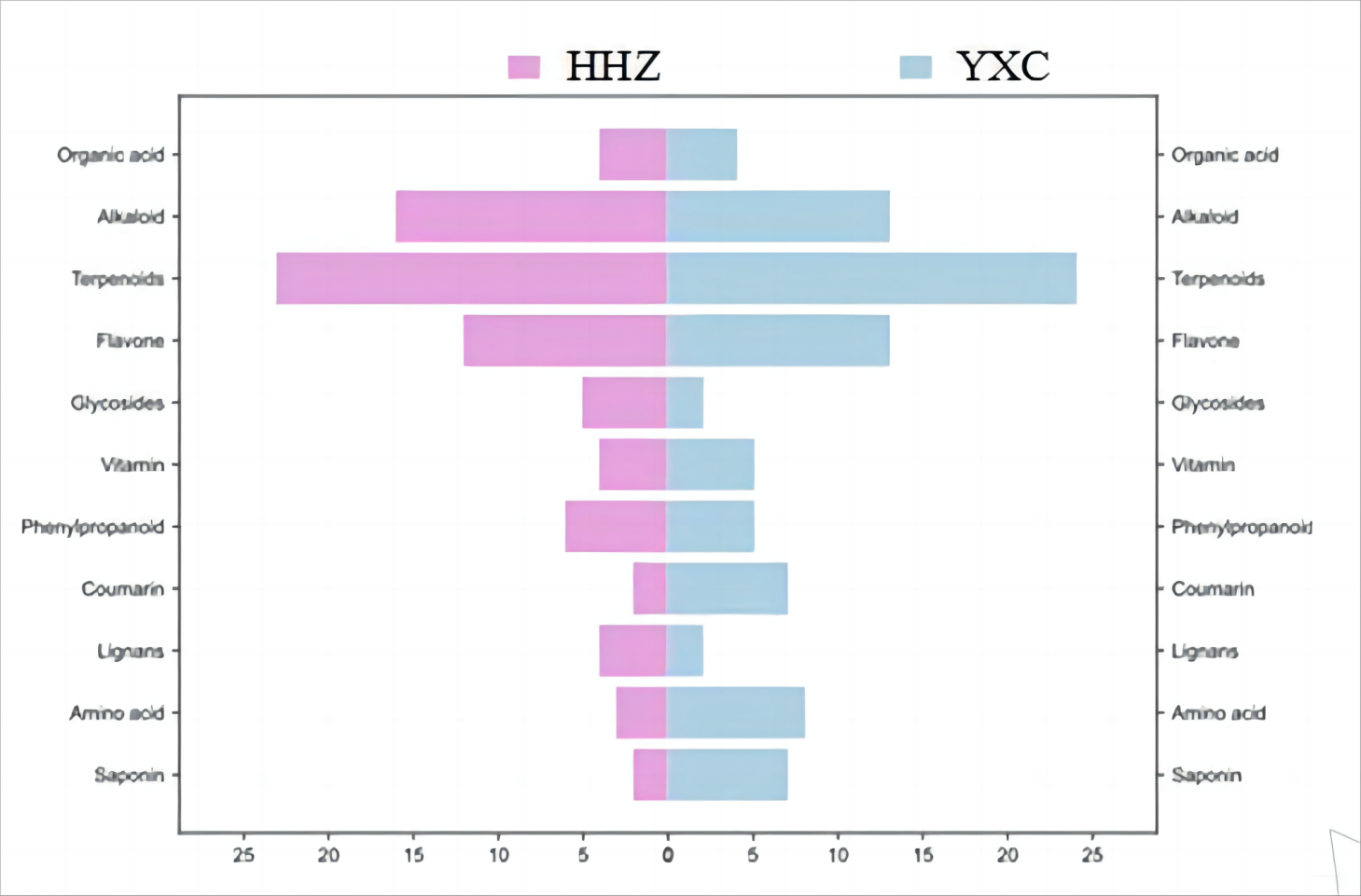


**Figure S2 Comparison of the relative contents of HHZ and YXC HHZ: *Sargassum pallidum* YXC：*Sargassum fusiforme***


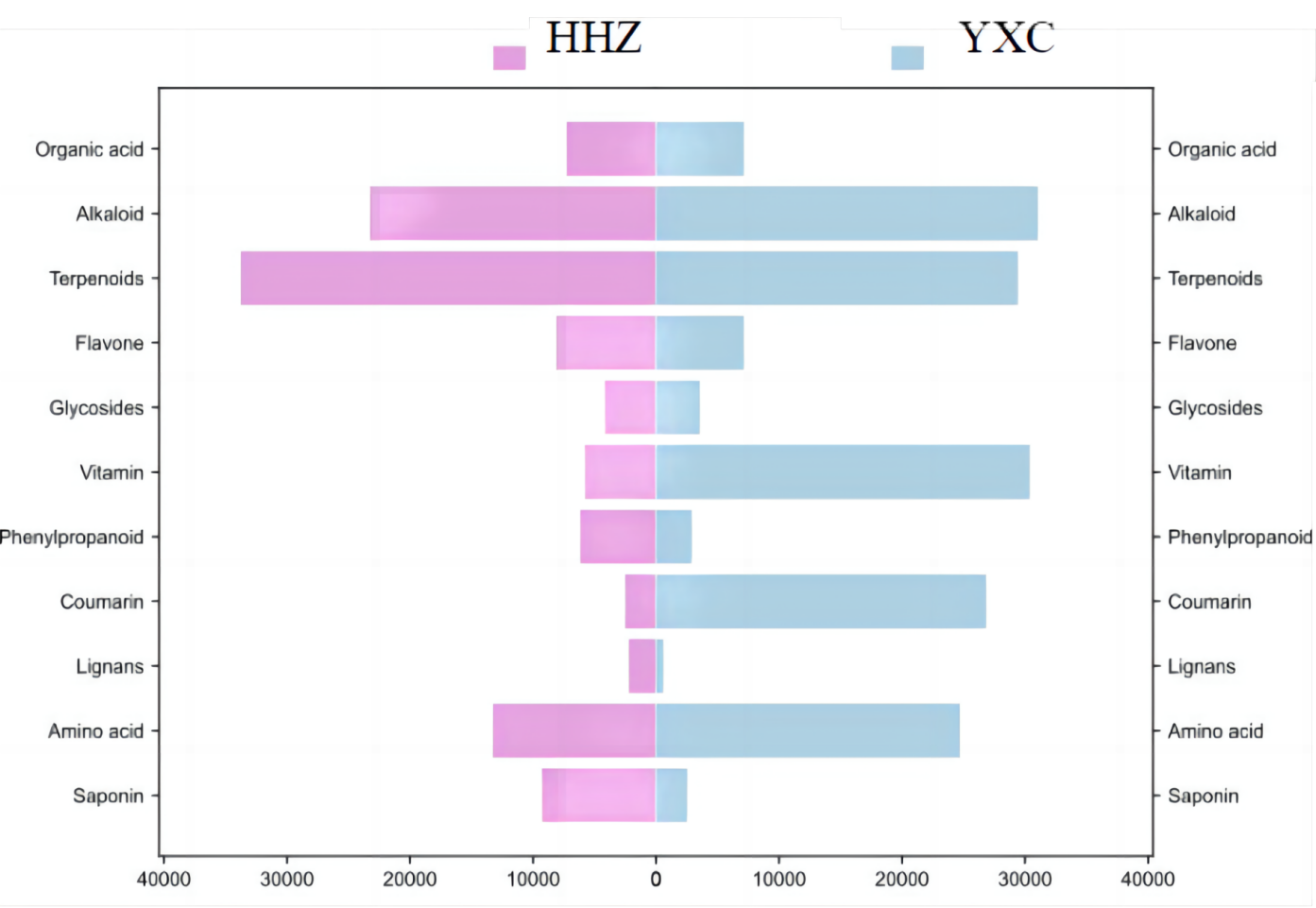


1. *Corresponding author: Tel/Fax: +86 791 8718099

   E-mail: zhugenhuajxzyy@163.com

   ^a^ These authors contributed equally to this work. [↑](#footnote-ref-1)
